# Cost-Effectiveness Analysis of Tyrosine Kinase Inhibitors in Gastrointestinal Stromal Tumor: A Systematic Review

**DOI:** 10.3389/fpubh.2021.768765

**Published:** 2022-01-10

**Authors:** Mingyang Feng, Yang Yang, Weiting Liao, Qiu Li

**Affiliations:** ^1^Department of Medical Oncology, Cancer Center, West China Hospital, Sichuan University, Chengdu, China; ^2^West China Biomedical Big Data Center, Sichuan University, Chengdu, China

**Keywords:** cost-effectiveness, economic evaluation, gastrointestinal stromal tumor, systematic review, TKI - tyrosine kinase inhibitor

## Abstract

**Background:** The introduction of tyrosine kinase inhibitor (TKI) therapy has dramatically improved the clinical effectiveness of patients with locally advanced and/or metastatic gastrointestinal stromal tumors (GIST), and this systematic review was conducted aiming at the cost-effectiveness analysis of TKIs in GIST.

**Methods:** A thorough literature search of online databases was performed, using appropriate terms such as “gastrointestinal stromal tumor or GIST,” “cost-effectiveness,” and “economic evaluation.” Data extraction was conducted independently by two authors, and completeness of reporting and quality of the evaluation were assessed. The systematic review was conducted following the PRISMA statement.

**Results:** Published between 2005 and 2020, 15 articles were incorporated into the systematic review. For advanced GIST, imatinib followed by sunitinib was considered cost-effective, and regorafenib was cost-effective compared with imatinib re-challenge therapy in the third-line treatment. For resectable GIST, 3-year adjuvant imatinib therapy represented a cost-effective treatment option. The precision medicine-assisted imatinib treatment was cost-effective compared with empirical treatment.

**Conclusion:** Although identified studies varied in predicted costs and quality-adjusted life years, there was general agreement in study conclusions. More cost-effectiveness analysis should be conducted regarding more TKIs that have been approved for the treatment of GIST.

**Systematic Review Registration:** https://www.crd.york.ac.uk/, PROSPERO: CRD42021225253.

## Introduction

Gastrointestinal stromal tumors (GIST) are rare mesenchymal tumors that predominantly originate from the gastrointestinal tract, mainly in the stomach (60%) and small intestine (30%) (1). Around 85% of GIST harbor gene mutations in stem cell factor receptor (KIT), and another 5–10% of GIST have a mutation in the gene encoding the platelet-derived growth factor receptors-α (PDGFRA) (2–5). Since the development and application of tyrosine kinase inhibitor (TKI) therapy that inhibits KIT and PDGFRA kinase activity and then intercepted the signal transduction pathways related to tumor proliferation and apoptosis, the therapeutic effects of locally advanced and/or metastatic GIST has achieved a revolutionary breakthrough.

The first TKI *imatinib mesylate* was approved in February 2002, for the treatment of KIT-positive metastatic and/or locally advanced GIST (6, 7). Treated with initial dose at 400 mg/day of imatinib, patients with metastatic or unresectable GIST reached median progression-free survival (mPFS) at 18 months, median overall survival (mOS) at 55 months (8–10). Other phase III studies have assessed the efficacy of imatinib at two initial dose levels (400 vs. 800 mg daily, given as 400 mg twice a day), showing equivalent response rates and OS for both dose levels (10–12). For resectable GIST patients, imatinib has been used in both pre- and post-operative therapy as several prospective studies have demonstrated the safety and efficacy of preoperative imatinib in patients undergoing surgical resection (13–15), while other studies revealed adjuvant imatinib therapy was associated with longer relapse-free survival (RFS) (16–18) and a longer duration (36- vs. 12-month group) of postoperative imatinib therapy improved RFS and OS for patients with a high risk of recurrence (19, 20).

Resistance to imatinib therapy is categorized into two situations. A small number (<15%) of patients have primary resistance to imatinib therapy (21), which is a disease that cannot be stabilized or progress within 6 months of initiation of treatment. The majority of patients (50%) develop secondary resistance characterized by an initial response or stable disease but subsequent progression, which is the result of acquired mutations generated during the course of treatment (22). For patients with imatinib-resistant or intolerant GIST, *sunitinib* was approved and recommended in January 2006, as it significantly improved median time to tumor progression (mTTP) (27.3 weeks in patients receiving sunitinib vs. 6.4 weeks in patients on placebo) and estimated OS (23). An recent study suggested that via sunitinib therapy, GIST patients after imatinib failure could reach the mTTP at 8.3 months and median mOS at 16.6 months (24).

In patients with metastatic or unresectable GIST progressing after the failure of imatinib and sunitinib, *regorafenib* was approved and regarded as the preferred option for third-line therapy, as it provided a significant improvement in PFS compared with placebo (4.8 months for regorafenib vs. 0.9 months for placebo) and higher disease control rate (DCR; 53 vs. 9%) (25).

Concerning rational decision making in health care, a major challenge in pharmacoeconomic evaluation is to make full use of cost-effectiveness data to optimize clinical practice and allocation of healthcare resources. This review was conducted aiming at the cost-effectiveness analysis of TKIs in GIST.

## Materials and Methods

This systematic review was conducted using the Preferred Reporting Items for Systematic Reviews and Meta-Analyses: The PRISMA Statement (26). PICOS criteria (population, intervention, control, outcomes, and study design) was used to guide the development of the search strategy. A thorough literature search of the following online databases was performed: PubMed, Web of Science, and Embase. Medical Subject Heading (MeSH) terms were individually selected using the National Library of Medicine controlled vocabulary thesaurus used for indexing articles: gastrointestinal stromal tumor or GIST, cost, cost-effectiveness, economic evaluation, economics, monetary, reimbursement, insurance. Searches were conducted on December 9, 2020 and all studies published before this date will be investigated.

Eligibility criteria were published studies in English evaluating the cost-effectiveness of any of the TKIs in GIST. Care was taken to ensure that the inclusion criteria were sufficiently broad so that possibly pertinent publications could be assessed by individual screening. Given the heterogeneity of available studies, we were not able to perform a meta-analysis.

Study data extraction was conducted independently by two authors (M.F., Y.Y.) and was extracted using a data extraction form, which included author, published year, country, study population, study design, intervention and comparison, model type, perspective, time horizon, discount rate, sensitivity analysis, threshold, sponsors, cost-effectiveness outcomes, and conclusions. To allow direct comparisons across countries, all costs were converted to US dollars, then inflated to December 2020 using the country-specific Consumer Price Index (CPI) (https://www.bls.gov/data/inflation_calculator.htm).

Completeness of reporting was assessed using the Consolidated Health Economic Evaluation Reporting Standards (CHEERS) checklist, which provides 24 items and accompanying recommendations to optimize reporting of health economic evaluations (27). The quality of the evaluation was assessed using the quality of health economic studies (QHES) instrument, which is designed to discriminate higher-quality cost-effectiveness information to enhance decision making (28). The QHES instrument was a quantitative and weighted scoring approach to appraise health economic evaluations, consisting of 16 items and each of them has a weighted point value ranging from 1 to 9. The sum of the weights of a study ranges between 0 (means extremely poor quality) and 100 (means excellent quality). Both checklists were completed independently by two authors (M.F., Y.Y.), and disagreements were resolved by discussion and arbitration (W.L.) where necessary.

This review has been registered with PROSPERO (CRD42021225253).

## Results

**Figure 1:**
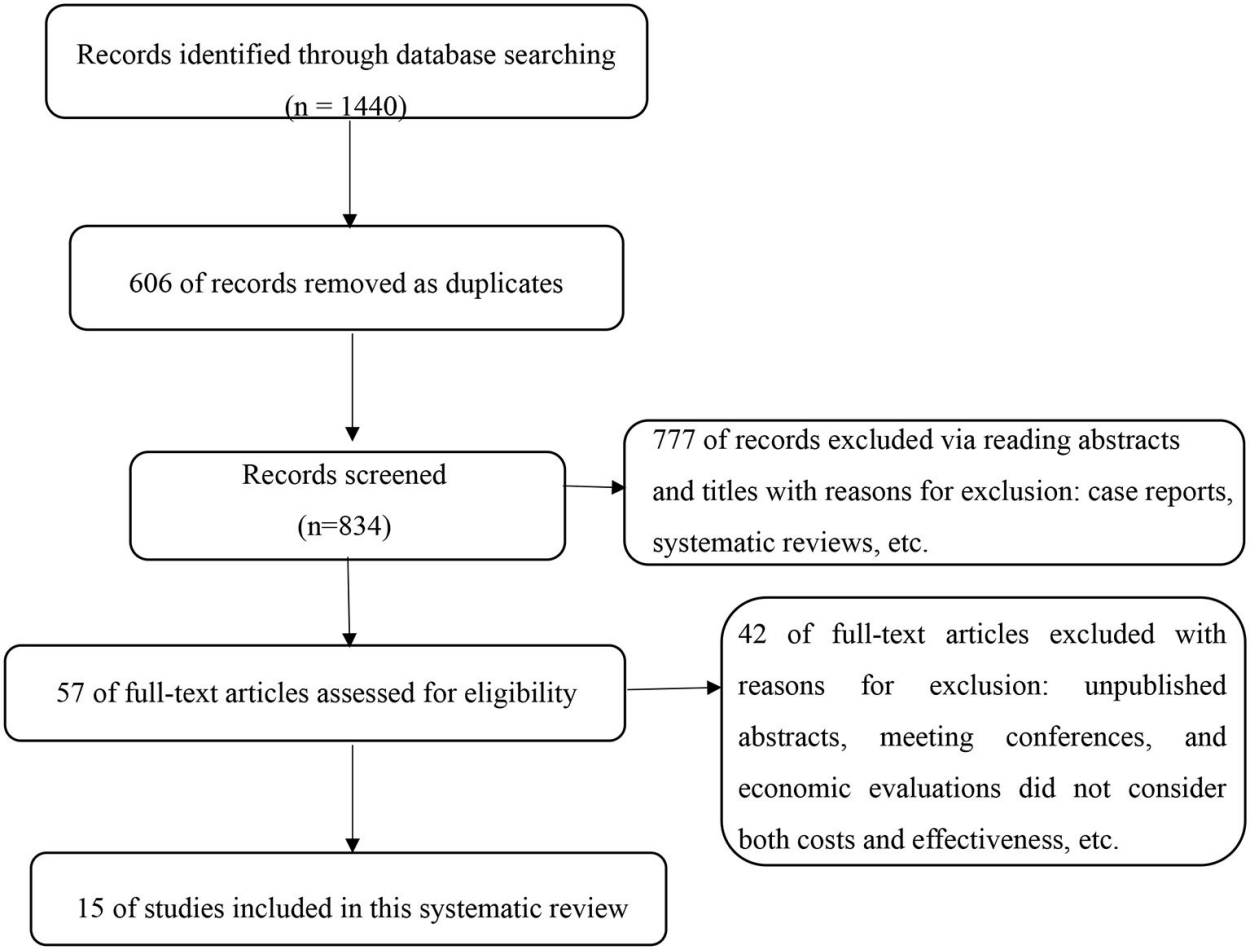
The process of selecting eligible articles for further research.

Based on the initial searches, a total of 1,440 articles were identified, which were independently screened by two reviewers (M.F., Y.Y.). Of these, 606 were removed as duplicates. Of the 834 publications remaining, 777 records were excluded via reading abstracts and titles with reasons for exclusion: case reports, reviews, and non-original research (e.g., letters or commentaries). Unpublished abstracts and meeting conferences were not included owing to the inability to completely assess quality. Then, 57 full-text articles were assessed for eligibility by two reviewers independently (M.F., Y.Y.). Disagreements were resolved by discussion and arbitration (W.L.) where necessary. Finally, 15 original investigations were found to have sufficient focus and relevance to be incorporated into the systematic review (Figure 1).

### Study Design and Structural Assumptions

The 15 identified studies were published between 2005 and 2020, Tables 1, 2 illustrates the general information, information of economic analysis, and outcomes and findings. Most studies were set in the European countries (*n* = 7), with three from the United States, two from Canada, and one each from Thailand, Mexico, and Singapore. Five studies were sponsored by the pharmaceutical industry (30, 32, 37, 40, 41), two declared there was no resources of funding (36, 43), four was funded independently (29, 35, 39, 42), and two did not include declarations of funding (31, 38). Besides, there were two study that did not specify the source of funding but the authors worked for pharmaceutical industry at the time of study (33, 34).

**Table 1:** Summary of included economic evaluations for advanced GIST.

**General information**				**Economic analysis**				**Outcomes and key findings**	
**Author, year, country, QHES score**	**Study population**	**Intervention**	**Comparator**	**Model type**	**Perspective, sponsor**	**Time horizon, discount rate, threshold**	**Sensitivity analysis**	**Cost effectiveness, 2020 US$**	**Conclusions**
Wilson (29), 2005, UK, 88	Unresectable and/or metastatic, KIT-positive GIST	IM 400 or 600 mg/day	BSC (Historical controls)	Two-state, three-state transition model, and four-state probability Markov model	UK NHS, NICE HTA programme	10 years, Costs: 6%, Benefits: 1.5%, NS	Sensitivity analysis, Monte Carlo simulation	2 years: $203,514/QALY; 5 years: $98,431/QALY; 10 years: $71,136/QALY	NS.
Huse (30), 2007, US, 89	Unresectable or metastatic GIST	IM 400 mg/day	Untreated (palliative and supportive care)	NS	US societal, Novartis Pharmaceuticals	10 years, 3%, $50,000/QALY	Sensitivity analysis	$51,619/QALY	IM 400 mg/day is cost-effective.
Mabasa (31), 2008, CA, 82	Advanced GIST	IM 400 mg/day, increased to 600–800 mg/day with PD	Historical controls	No economic model was used	BCCA, NS	NA, 3 and 5%, $50,000/QALY	Sensitivity analysis	$18,293/LYG	IM for advanced GIST seems cost-effective.
Chabot (32), 2008, CA, 89	Unresectable or metastatic GIST intolerant or resistant to IM	SU plus BSC	Placebo plus BSC	Markov model	Provincial health ministry, Pfizer Canada Inc.	Lifetime, 5%, $132,166/QALY	Sensitivity analysis	$86,900/QALY $54,202/LYG	SU is cost-effective for patients with unresectable, recurrent, or metastatic GIST and have failed or are intolerant to IM.
Paz-Ares (33), 2008, Spain, 93	Unresectable or metastatic GIST intolerant or resistant to IM	SU plus BSC	Placebo plus BSC	Markov three-state	Spanish National Health System, NS	6 years, 3.5%, $50,000/QALY	Sensitivity analysis, Monte Carlo simulation	$83,094/QALY $51,190/LYG	SU should be considered a cost-effective alternative for the second-line treatment of GIST.
Contreras-Hernande (34), 2008, Mexico, 97	Advanced GIST	High dose IM 800 mg/day or SU	Palliative care	Markov three-state	IMSS, NS	5 years, 5%, $51,300/QALY	Sensitivity analysis, Monte Carlo simulation	SU vs. palliative care, $54,601/LYG; SU vs. high dose IM, dominant	SU would be cost-effective in second-line treatment.
Hislop (35), 2011, UK, 96	Unresectable and/or metastatic GISTs progressed on treatment with IM at 400 mg/day or intolerant to IM	Path-2 IM 600–800 mg to SU; Path-3 IM 600 mg to SU; Path-4 IM 600 mg; Path-5 IM 800 mg to SU; Path-6 IM 800 mg; Path-7 SU	Path-1 BSC	Markov model	UK NHS, NICE HTA programme	10 years, 3.5%, variable threshold	Sensitivity analysis, Monte Carlo simulation	Path-1: reference; Path-7: $545,724/QALY; Path-4: $54,708/QALY; Path-3: $143,708/QALY; Path-6: dominated; Path-5: dominated; Path-2: $88,880/QALY	If society's WTP is ~£25,000/QALY, BSC is cost-effective; when WTP is £25,000–£45,000/QALY, IM 600 mg/d is cost-effective; when WTP is £45,000/QALY~, IM 600 mg/d to IM 800 mg/d to SU is cost-effective.
Nerich (36), 2016, France, 96	Advanced GIST	Strategy 2: IM 400 mg/day–IM 800 mg/day-BSC; Strategy 3: IM 400 mg/day-SU-BSC; Strategy 4: IM 400 mg/day–IM 800 mg/day-SU-BSC	Strategy 1: IM 400 mg/day-BSC	Markov decision-analysis model	French Public Healthcare System, None	Lifetime, 4%, €50,000/LYG	Sensitivity analysis, Monte Carlo simulation	S3 vs. S1: $72,096/LYG; S2 vs. S3: dominated; S4 vs. S3: $542,574/LYG	IM in first-line treatment, followed by SU in second-line treatment strategy may be considered as the best cost-effective strategy.
Tamoschus (37), 2017, Germany, 100	Unresectable or metastatic GIST patients who have progressed on, or are intolerant or resistant to IM and SU	Regorafenib 160 mg/day	IM rechallenge 400 mg/day	Partitioned survival model	German payer, Bayer Pharmaceuticals	Lifetime, 3.5%, €50,000/QALY	Sensitivity analysis, Monte Carlo simulation	$25,394/QALY $17,229/LYG	Regorafenib is cost-effective compared with IM rechallenge in Germany.
Zuidema (38), 2019, Netherlands, 93	Unresectable or metastatic GIST	TDM-guided dosing IM	Fixed dosing IM	Partitioned survival model	The societal perspective, NS	5 years, costs: 4%, benefits: 1.5%, €80,000/QALY	Sensitivity analysis, Monte Carlo simulation	$71,453/QALY $67,756/LYG	TDM-guided dosing may be a cost-effective intervention.
Banerjee (39), 2020, US, 96	Metastatic GIST	TGT- and variation-directed first-line therapy: KIT exon 9 variations: high-dose IM-SU-BSC	Empirical imatinib therapy (IM 400 mg-IM 800 mg-SU-BSC)	Markov model	US payer perspective, Surgical Society of the Alimentary Tract Mentored Research Award	10 years, 3%, $100,000/QALY	Sensitivity analysis, Monte Carlo simulation	$93,501/QALY	TGT-directed therapy is cost-effective compared to empirical IM.

*QHES, quality of health economic studies; GIST, gastrointestinal stromal tumors; BSC, best supportive care; NHS, national health service; NICE, national institute for health and clinical excellence; HTA, health technology assessment; IM, imatinib; NS, not specified; QALY, quality-adjusted life-year; PD, progressive disease; BCCA, British Columbia Cancer Agency; LYG, life year gained; SU, sunitinib; IMSS, Instituto Mexicano del Seguro Social; WTP, willingness to pay; TDM, Therapeutic drug monitoring; TGT, targeted gene testing*.

**Table 2:** Summary of included economic evaluations for resectable GIST.

**General information**				**Economic analysis**				**Outcomes and key findings**	
**Author, year, country, QHES score**	**Study population**	**Intervention**	**Comparator**	**Model type**	**Perspective, sponsor**	**Time horizon, discount rate, threshold**	**Sensitivity analysis**	**Cost effectiveness, 2020 US$**	**Conclusions**
Sanon (40), 2013, US, 96	Resected primary GIST	3-year adjuvant IM 400 mg/day	1-year adjuvant IM 400 mg/day	Markov 3-state	A third party payer, Novartis Pharmaceuticals	Lifetime, 3%, $100,000/QALY	Sensitivity analysis, Monte Carlo simulation	$74,792/QALY $68,102/LYG	Treating surgically resected GIST patients with 3 years adjuvant IM is cost-effective.
Majer (41), 2013, Netherlands, 100	Resected primary GIST patients who have high risks of tumor recurrence	3-year adjuvant IM 400 mg/day	1-year adjuvant IM 400 mg/day	Multistate Markov model	Dutch healthcare provider, Novartis Oncology	Lifetime, costs: 4%, benefits: 1.5%, €50,000/QALY	Sensitivity analysis, Monte Carlo simulation	$49,894/QALY $36,520/LYG	Longer-term (3 years) adjuvant IM therapy represents a cost-effective treatment option.
Bussabawalai (42), 2019, Thailand, 96	Localized GIST patients who underwent complete resections and had a high risk of recurrence	Option 2: Recurrence during therapy: BSC; after therapy: IM 400 mg/day-BSC; 2.1: adjuvant IM 400 mg/day for 1 year; 2.2: for 3 years; Option 3: Recurrence during therapy: SU-BSC; after therapy: IM 400 mg/day-SU-BSC; 3.1: adjuvant IM 400 mg/day for 1 year; 3.2: for 3 years; Option 4: No adjuvant IM-IM 400 mg/day-SU-BSC	Option 1: No adjuvant IM-IM 400 mg/day-BSC	Markov 3-state	The societal perspective, National Health Security Office	Lifetime, 3%, 160,000 THB/QALY	Sensitivity analysis, Monte Carlo simulation	Option 2.1, 3.1, 4 were dominated by 2.2; Option 2.2 vs. 1: $55,463/QALY; Option 3.2 vs. 2.2: $87,737/QALY	Adjuvant IM treatment improved the health benefits of patients with high risk of GIST recurrence. However, in the Thai context, it was not cost-effective at the current price.
Farid (43), 2020, Singapore, 96	Rectal GIST patients requiring abdominoperineal resection following neoadjuvant IM	UAPR	CIUP	Markov decision model	Healthcare payers' perspective, None	20 years, 3%, 50,000 SGD/QALY	Sensitivity analysis, Monte Carlo simulation	UAPR dominates CIUP being both more effective (8.66 QALYS vs 5.43 QALYs) and less expensive ($241,499 vs $261,881).	UAPR is more effective and less costly than CIUP.

*QHES, quality of health economic studies; GIST, gastrointestinal stromal tumors; IM, imatinib; QALY, quality-adjusted life-year; LYG, life year gained; BSC, best supportive care; SU, sunitinib; UAPR, upfront abdominoperineal resection; CIUP, continued IM until progression*.

Most studies (*n* = 8) used a Markov modeling approach (32–35, 39–42). Two study used a Markov decision-analysis model (36, 43), two used a partitioned survival model (37, 38), and the modeling approach was not clearly specified in one study (30). Five study used the conventional three-health state model of PFS, progressive disease, and death (33, 34, 37, 40, 42). One study determined seven clinically plausible pathways based on three-state model structure (35). One study encompassed four-health states: free of recurrence, first recurrence, second recurrence, and death (41). Another study constructed the model that simulated treatment outcomes following the treatment algorithm defined by the National Comprehensive Cancer Network (NCCN) guideline (39). One study used modified Novartis model, which contained two- and three-state transition model, and four-state probability Markov model (29). Another study performed a retrospective medical record review without applying any model (31).

The perspective of institution or healthcare system was most common (*n* = 7) (29, 31–33, 35, 36, 41), while one of them merely include the cost of drug acquisition, supply and labor and did not include surgery or radiotherapy costs, health care visits, or costs related to supportive care or adverse events (AEs) (31). Five studies were performed from the healthcare payer's perspective (34, 37, 39, 40, 43). Three studies claimed they provided the societal perspective (30, 38, 42), whereas two of them did not include indirect costs in the analysis (30, 38) and should be classified as healthcare system's perspective instead.

Varied from 5 years to lifetime, time horizons were clearly specified in most studies (*n* = 14), except in the one that was a retrospective review (31). Time horizons were put in sensitivity analysis in six studies (30, 34, 35, 37, 39, 41). Lifetime was the most frequently used time horizon option (*n* = 6) (32, 36, 37, 40–42).

All studies specified a discount rate in their analysis. The discount rates of cost varied from 3 to 6% and benefits varied from 1.5 to 5%. Three studies applied different discount rates to costs and benefits (29, 38, 41) and the remaining studies applied the same rate to both costs and outcomes.

Four studies estimated model costs in USD (30, 34, 39, 40), two each in GBP (29, 35) and CAD (31, 32), five in EUR (33, 36–38, 41), and one each in THB (42) and SGD (43). Threshold was specified in most studies (*n* = 14).

Most studies focused on cost-effectiveness of TKIs used in patients with unresectable and/or metastatic GIST (*n* = 11) (29–39). Three studies focused on cost-effectiveness of adjuvant imatinib therapy after resection (40–42). Another study focused on rectal GIST patients requiring abdominoperineal resection following neoadjuvant imatinib (43).

Five studies used evidence from a single phase II/III clinical trial and include only one comparator (30, 32, 33, 40, 41). For the remaining studies, approaches to evidence synthesis were varied and included a systematic review to identify clinical inputs (29, 35, 36, 42), from previously published studies (38, 39, 43), comparison between uncontrolled trials and historical control patients (29), Bucher indirect comparison (37), comparisons via reviewing retrospective medical record (31, 34), and comparison between two RCTs by using the indirect treatment comparison program developed by the Canadian Agency for Drugs and Technologies in Health (CADTH) (42).

Most commonly, PFS and OS outcomes from clinical trials were the source of treatment effects in the studies, while one study also used the data of time to treatment failure (TTF) (29). In most cases, it was necessary to extrapolate the data to the time horizon of the model, except in a pragmatic, population-based review (31). Parametric extrapolation methods were the most common, and two studies had used several extrapolation methods, including Gompertz, Weibull, and log-logistic, and chose the best fitted parametric model (37, 41). Transition probabilities were calculated using the Declining Exponential Approximation of Life Expectancy (DEALE) method in another study (36), which is an approximation of life expectancy by using a simple exponential function for survival. Extrapolation of OS curves used external data sources [i.e., retrospective studies or databases like Surveillance, Epidemiology, and End Results (SEER)] in some studies to simulate the natural disease history (35, 38–40, 43). In addition, patients' data in the real world were collected in several studies (30, 42), due to the lack of clinical or cost data.

Most identified studies (*n* = 12) were cost-utility analyses. Utility values were sourced from a mapping of Eastern Cooperative Oncology Group (ECOG) performance status from pivotal clinical trials to EuroQol-5 Dimensions (EQ-5D) scores (29, 30), obtained from EQ-5D scores directly collected in clinical trials (32, 33, 37), comprehensively extracted from previously published economic evaluations (35, 38–41, 43), or use the EQ-5D-3L questionnaire to interview local hospital's patients and convert the quality of life scores into utility values (42). Two studies applied a utility improvement during the treatment off period (32, 33), and two studies applied a utility decrement for AEs (40, 41), while one study claimed that aggregate utility values had already included any disutilities associated with AEs (37).

The estimation of costs varied in the studies. Drug acquisition costs mostly come from public institutional databases, except for one study that drug was not available in the market at the time of the analysis, so its cost information was provided by pharmaceutical manufacturer (34). Management of AEs related costs were calculated in several studies (*n* = 8) (29, 32, 33, 35, 38, 40–42), while one study only include direct drug acquisition costs (37). Costs of genetic testing were included in two studies (36, 39). Costs of other cancer types (i.e., pancreatic cancer and ovarian cancer) were used as models to estimate the costs of medical management due to the lack of GIST cost data in two studies (30, 33). End-of-life costs were included in only one study (32).

### Model Outcomes

#### TKIs in Advanced GIST

Imatinib was firstly compared with best supportive care (BSC) or historical controls in unresectable and/or metastatic, KIT-positive GIST in three studies (29–31), and was associated with an increase in costs and QALYs compared to BSC in all studies. The predicted QALYs associated with imatinib varied from 4.15 QALYs (30) to 4.85 QALYs (29) in 10 years' time horizon, while a retrospective medical record indicated that imatinib therapy was associated with 5.56 life years gained (LYGs) (31). The predicted total costs ranged from $91,950 (31) to $554,880 (30). In the earliest economic analysis of imatinib we included, the authors calculated incremental cost-effectiveness ratio (ICER) in different time horizons at $203,514/QALY (2 years), $98,431/QALY (5 years), and $71,136/QALY (10 years), respectively (29) in UK, claiming that the estimates after 2 years were of great uncertainty because they were based on the extrapolation beyond the trial data. Another study calculated ICER at $51,619/QALY, and concluded that the findings suggested imatinib was cost-effective in the US according to NCCN guidelines (30), the other study calculated ICER at $18,293/LYG and concluded that imatinib seemed cost-effective at willingness-to-pay (WTP) threshold of $50,000/QALY in Canada (31).

For unresectable or metastatic GIST patients who were intolerant or resistant to imatinib, sunitinib was compared with BSC in two studies (32, 33) based on the results of the pivotal phase III trial (23), and both studies predicted that sunitinib was associated with an increase in costs and QALYs and were likely to be cost-effective at the WTP thresholds. They were associated with costs ranging from $39,370 (33) to $50,176 (32) and QALYs ranging from 0.97 QALYs (32) to 1.00 QALYs (33), resulting in ICER at $86,900/QALY (32) and $83,094/QALY (33), respectively. For patients who were intolerant or resistant to both imatinib and sunitinib, regorafenib ($26,566, 1.691 QALYs) was compared with imatinib re-challenge therapy ($16,021, 1.275 QALYs) using a partitioned survival model, resulting in ICER at $25,394/QALY and was thought to be cost-effective in Germany (37).

Several other articles have constructed a variety of treatment pathways to carry out an economic evaluation of treatment methods for advanced GIST. One study compared high-dose imatinib, sunitinib, and BSC in the second-line treatment of advanced GIST (34). In this study, sunitinib was dominant of high-dose imatinib, because it costed less ($21,085 vs. $41,713) and produced more effectiveness (1.4 LYGs vs. 1.31 LYGs). Compared with BSC, sunitinib was associated with an ICER of $54,601/LYG and was considered the most cost-effective option. Another study constructed seven clinical treatment pathways for advanced GIST patients who had progressed on treatment with regular-dose imatinib or were intolerant to imatinib (35). Total costs ranged from $185,961 to $344,932 and QALYs ranged from 2.397 QALYs to 4.803 QALYs among the seven pathways. The BSC was considered as the most cost-effective when WTP was under £25,000/QALY, while imatinib 600 mg/day was the most cost-effective when WTP was during £25,000–£45,000/QALY and “imatinib 600 mg/day followed by imatinib 800 mg/day followed by sunitinib” was the most cost-effective when WTP was above £45,000/QALY. Similarly, another study constructed four clinical treatment pathways using the Markov decision-analysis model and concluded imatinib 400 mg/day in first-line treatment, followed by sunitinib in second-line treatment strategy may be considered as the best cost-effective strategy (36).

The cost-effectiveness of therapeutic drug monitoring (TDM) guided dosing imatinib was investigated in comparison with fixed dosing imatinib (38). The TDM-guided dosing imatinib was associated with an increase in costs ($182,901 vs. $130,050) and QALYs (3.54 QALYs vs. 2.80 QALYs) compared with fixed dosing imatinib, producing an ICER at $71,453/QALY which was considered cost-effective. Another study (39) assessed the cost-effectiveness of targeted gene testing (TGT) directed therapy (TGT means if KIT exon 9 variations is positive, then directly use imatinib 800 mg/day) was compared with empirical therapy (imatinib 400 mg/day to imatinib 800 mg/day to sunitinib to BSC). The TGT-directed therapy was associated with an increase in cost, from $476,242 with the empirical imatinib approach to $485,900 with TGT-directed therapy. QALYs increased by 0.10, from 4.88 with empirical imatinib to 4.98 with TGT-directed therapy, so TGT-directed therapy yielded an ICER of $93,501/QALY which was considered cost-effective at a WTP threshold of $100,000/QALY.

#### TKIs in Resectable GIST

For patients with resected primary GIST, the cost-effectiveness of 1- vs. 3-year adjuvant imatinib 400 mg/day treatment after resection was compared in two studies (40, 41) based on the data of SSGXVIII/AIO clinical trial (19). They found that 3-year adjuvant therapy was associated with increased costs and QALYs, thus resulting in ICER at $74,792/QALY (40) and $49,894/QALY (41), respectively. Both studies concluded that 3-year adjuvant therapy was a cost-effective treatment option under the WTP threshold.

For patients with resected localized GIST and had a high risk of recurrence, clinical treatment pathways of four alternative treatment options were constructed (42). In the study, option 2.2 (adjuvant imatinib 400 mg/day for 3 years) was most likely to be the cost-effective option as it was dominant to other three options, but was not cost-effective at the current price in the authors' country. Another economic evaluation (43) was conducted from a novel perspective: for rectal GIST patients requiring abdominoperineal resection following neoadjuvant imatinib, upfront abdominoperineal resection (UAPR) was compared with continued imatinib until progression (CIUP). The author concluded that UAPR dominates CIUP for being more effective (8.66 QALYS vs. 5.43 QALYs) and less expensive ($241,499 vs. $261,881).

### Reporting and Quality Assessment

The CHEERS checklist was used to review completeness of reporting of the evaluation. Compliance with the CHEERS checklist was variable. Two studies were found to have perfect compliance with the CHEERS reporting requirements (37, 42). Seven studies were assessed as having only one non-compliance (29, 35, 36, 39–41, 43), two each were found to have two non-compliances (33, 38), three non-compliances (30, 32), and four non-compliances (31, 34). Many studies (*n* = 7) did not describe the population and methods used to elicit preferences for outcomes. Most studies (*n* = 13) reported the dates of the estimated resource quantities and unit costs and described methods for converting costs into a common currency, except in two studies (29, 34).

The QHES instrument was used to assess of the quality of the economic evaluation. The mean QHES score was 93.8 ± 4.9 (range 82–100). Two studies were found to have perfect compliance with the QHES instrument (37, 41). Most studies (*n* = 11) did not clearly state the reason why the perspective of the analysis were chosen. Systematic reviews and quality assessment were performed in only three studies (35, 36, 42).

The complete tables of the CHEERS checklist and QHES instrument could be found in Supplementary Materials.

## Discussion

Almost every new drug is associated with better clinical benefits in patients and higher costs, posing challenges to cost-effectiveness and affordability, and results of economic evaluations have become increasingly important as criteria for the allocation of health care resources. In our study, there were major differences in the structural assumptions in the identified studies, including in the model types, study perspectives, time horizons, discount rates, assumption of utility, and extrapolation of survival. Therefore, there were large variations in the predicted costs and QALYs associated with each treatment, for example, the predicted QALYs of advanced GIST treated with imatinib varied from 2.96 to 4.85. Variations in QALYs could be explained by the use of utility values derived by different methods, different time horizons, and alternative approaches to survival extrapolation. Variations in total costs could be explained by different healthcare resource use and costs across jurisdictions. Moreover, the different study perspectives would significantly affect total costs. It may also be accounted for by different approaches to capturing costs of post-progression treatment, where some studies assumed no post-progression drug costs while others (35, 36, 42) constructed a series of pragmatic clinical treatment pathways and clearly calculated the costs of each treatment path.

Despite these variations, there was consistency in the conclusions across most of the studies. For patients with advanced/metastatic GIST, all publications agree that TKIs are associated with higher costs and effectiveness than placebo or empirical treatment. Some articles (29–31) concluded that imatinib 400 mg/d in first-line therapy was cost-effective, but these economic analyses were carried on between 2005 and 2008, and some model parameters they used may not be fully standardized. Other studies confirmed the cost-effectiveness of sunitinib in second-line therapy (32–34), and regorafenib was cost-effective compared with imatinib re-challenge in the third-line therapy in Germany (37). Two other studies simulated the most cost-effective medication plan by constructing multiple clinical pathways (35, 36), and based on these results, we suggest for advanced GIST, the treatment of imatinib in first-line, followed by sunitinib in second-line, and regorafenib in third-line was cost-effective.

For patients with resectable GIST, several studies (40, 41), respectively, investigated the 3- vs. 1-year adjuvant imatinib therapy in resected GIST, and both confirmed the cost-effectiveness of the longer-term (3-year) therapy. Another study (43) illustrates the necessity of surgery in rectal GIST patients requiring abdominoperineal resection following neoadjuvant imatinib. Most of the identified studies were conducted in high-income and developed countries, including European and American countries, and most studies had positive conclusions regarding the cost-effectiveness of the interventions except one study (42) taking into account the country's context.

Another two recent economic evaluations carried out by Banerjee et al. (39) and Zuidema et al. (38), respectively, are not limited to a fixed-dose of medication but are concerned about individualized medication methods that guide the use of TKIs in advanced GIST, such as TDM (38) and TGT (39), which are both considered cost-effective. It is known that mutational status has a dramatic impact on response to imatinib or sunitinib in patients with advanced or metastatic GIST. The presence of a KIT exon 11 mutation was associated with better response rates, PFS, and OS compared to KIT exon 9 mutations or wild-type GIST (44–46). In patients whose tumors expressed a KIT exon 9 mutation, high-dose imatinib (800 mg/d) resulted in a significantly superior PFS (44, 45) and increased response rates (46, 47) compared to those treated with imatinib 400 mg/d. And the cost-effectiveness analysis (39) focusing on TGT-guided therapy was performed based on this setting. Another study (38) focused on the TDM-guided dosing imatinib. Therapeutic drug monitoring is a technique used to determine the plasma exposure levels of certain drugs and enable to ensure the GIST patients redistributed with adequate imatinib concentrations in plasma (48, 49). By performing an economic evaluation between TDM-guided and fixed-dose imatinib, the results are a valuable addition to the investigation of the effect of dose optimization. It is foreseeable that with the further development of molecular oncology, there would be more novel economic evaluations.

At the same time, there existed other new TKIs that have been approved by the food and drug administration (FDA) and endorsed by NCCN guidelines, for instance, avapritinib for PDGFRA D842V-mutant GIST as first-line therapy (50), and ripretinib for the progressive disease after imatinib, sunitinib, and regorafenib as fourth-line therapy (51). Nevertheless, sorafenib, nilotinib, dasatinib, and pazopanib have also shown activity in patients with GIST resistant to imatinib and sunitinib. However, much of the data on these TKIs came from phase II studies or retrospective analyses, which lack high-quality clinical evidence. The cost-effectiveness of these TKIs still needs to be measured.

There exist some limitations in this study. First, the QHES instrument employs yes or no responses rather than a continuous scale for each criterion, which would lead to inaccuracy when a study actually partly meets the criteria but is appraised with zero points. Therefore, the CHEERS checklist was applied to cross-evaluate the quality of the literature. But the CHEERS statement is an assessment of reporting, not methodological quality, and failure to follow all the requirements in the CHEERS statement is not indicative of a poor-quality study. Second, our systematic review excluded conference abstracts, unpublished studies (gray literature), and studies that lack full-text resources, which may also introduce some bias.

In conclusion, our systematic review identified 15 economic evaluations of TKIs used in patients with GIST and demonstrated several important findings. First, for patients with advanced GIST, imatinib in the first-line treatment, followed by sunitinib in the second-line treatment was considered cost-effective, and regorafenib was cost-effective compared with imatinib re-challenge in the third-line therapy. Second, for patients with resectable GIST, 3-year adjuvant imatinib therapy represented a cost-effective treatment option compared with 1-year therapy. Third, the precision medicine-assisted imatinib treatment plan represented by TDM- and TGT-guided imatinib therapy was cost-effective compared with empirical fixed-dose treatment.

## Data Availability Statement

The original contributions presented in the study are included in the article/Supplementary Material, further inquiries can be directed to the corresponding author/s.

## Author Contributions

MF: material preparation, data acquisition, and wrote the first draft of the manuscript. YY: material preparation and data acquisition. The revised draft of the manuscript was written by MF and YY. All authors contributed to the conception and design of the study, commented on previous versions of the manuscript, read, and approved the final manuscript.

## Funding

This work was supported by the 1.3.5 Project for Disciplines of Excellence, West China Hospital, Sichuan University (grant No. ZYJC18008 and No. ZYJC18010). The funding source was not involved in the study design, data collection, data analysis, data interpretation, the writing of this article or the decision to submit the paper for publication.

## Conflict of Interest

The authors declare that the research was conducted in the absence of any commercial or financial relationships that could be construed as a potential conflict of interest.

## Publisher's Note

All claims expressed in this article are solely those of the authors and do not necessarily represent those of their affiliated organizations, or those of the publisher, the editors and the reviewers. Any product that may be evaluated in this article, or claim that may be made by its manufacturer, is not guaranteed or endorsed by the publisher.
