# Supplementary material for: Cost-Effectiveness Analysis of Tyrosine Kinase Inhibitors in Gastrointestinal Stromal Tumor: A Systematic Review
Source: Front Public Health. 2022 Jan 10;9:768765. doi: 10.3389/fpubh.2021.768765 (PMC8784780; doi:10.3389/fpubh.2021.768765)
Supplement: Supplementary file 1 [file Table_1.DOCX]

**Supplementary tables**

Cost-effectiveness analysis of tyrosine kinase inhibitors in gastrointestinal stromal tumor: a systematic review

Mingyang Feng^1,2^; Yang Yang^1,2^; Weiting Liao^1,2^; Qiu Li^1,2^

1: Department of Medical Oncology, Cancer Center, West China Hospital, Sichuan University, Chengdu 610041, China; 2: West China Biomedical Big Data Center, Sichuan University, Chengdu 610041, China;

**Correspondence:** Qiu Li, Department of Medical Oncology, Cancer Center, West China Hospital, Sichuan University, No. 37, GuoXue Xiang, Chengdu 610041, China, email: fbqiu9@163.com; Tel: + 86 28-85423262; Fax: + 86 28-85423609.

**Table 1. Summary of included economic evaluations (see document in xlsx.).**

**Table 2. Review completeness of reporting of studies with CHEERS checklist.**

**Table 3. Quality assessment of studies with QHES instrument.**

Table 2. Review completeness of reporting of studies with CHEERS checklist.

|  | Item 1 | Item 2 | Item 3 | Item 4 | Item 5 | Item 6 | Item 7 | Item 8 | Item 9 | Item 10 | Item 11 | Item 12 | Item 13 | Item 14 | Item 15 | Item 16 | Item 17 | Item 18 | Item 19 | Item 20 | Item 21 | Item 22 | Item 23 | Item 24 | Number of compliances |
| --- | --- | --- | --- | --- | --- | --- | --- | --- | --- | --- | --- | --- | --- | --- | --- | --- | --- | --- | --- | --- | --- | --- | --- | --- | --- |
| Wilson, 2005 | √ | √ | √ | √ | √ | √ | √ | √ | √ | √ | √ | √ | √ | × | √ | √ | √ | √ | √ | √ | √ | √ | √ | √ | 23 |
| Huse, 2007 | √ | × | √ | √ | √ | √ | √ | √ | √ | √ | √ | × | √ | √ | × | √ | √ | √ | √ | √ | √ | √ | √ | √ | 21 |
| Mabasa, 2008 | √ | √ | √ | √ | √ | √ | √ | × | √ | √ | √ | NA | √ | √ | × | √ | √ | √ | √ | √ | √ | √ | × | √ | 20 |
| Chabot, 2008 | × | × | √ | √ | √ | √ | √ | √ | √ | √ | √ | √ | √ | √ | × | √ | √ | √ | √ | √ | √ | √ | √ | √ | 21 |
| Paz-Ares, 2008 | √ | √ | √ | √ | √ | √ | √ | √ | √ | √ | √ | √ | √ | √ | √ | √ | √ | √ | √ | √ | √ | √ | × | × | 22 |
| Contreras-Hernande, 2008 | √ | × | √ | √ | √ | √ | √ | √ | √ | √ | √ | NA | √ | × | √ | √ | √ | √ | √ | √ | √ | √ | × | √ | 20 |
| Hislop, 2011 | √ | √ | √ | √ | √ | √ | √ | √ | √ | √ | √ | × | √ | √ | √ | √ | √ | √ | √ | √ | √ | √ | √ | √ | 23 |
| Sanon, 2013 | √ | √ | √ | √ | √ | √ | √ | √ | √ | √ | √ | × | √ | √ | √ | √ | √ | √ | √ | √ | √ | √ | √ | √ | 23 |
| Majer, 2013 | √ | √ | √ | √ | √ | √ | √ | √ | √ | √ | √ | × | √ | √ | √ | √ | √ | √ | √ | √ | √ | √ | √ | √ | 23 |
| Nerich, 2016 | √ | √ | √ | √ | √ | √ | √ | √ | √ | √ | √ | NA | √ | √ | √ | √ | √ | √ | √ | √ | √ | √ | √ | √ | 23 |
| Tamoschus, 2017 | √ | √ | √ | √ | √ | √ | √ | √ | √ | √ | √ | √ | √ | √ | √ | √ | √ | √ | √ | √ | √ | √ | √ | √ | 24 |
| Bussabawalai, 2019 | √ | √ | √ | √ | √ | √ | √ | √ | √ | √ | √ | √ | √ | √ | √ | √ | √ | √ | √ | √ | √ | √ | √ | √ | 24 |
| Zuidema, 2019 | √ | √ | √ | √ | √ | √ | √ | √ | √ | √ | √ | × | √ | √ | √ | √ | √ | √ | √ | √ | √ | √ | × | √ | 22 |
| Farid, 2020 | √ | √ | √ | √ | √ | √ | √ | √ | √ | √ | √ | × | √ | √ | √ | √ | √ | √ | √ | √ | √ | √ | √ | √ | 23 |
| Banerjee, 2020 | √ | √ | √ | √ | √ | √ | √ | √ | √ | √ | √ | × | √ | √ | √ | √ | √ | √ | √ | √ | √ | √ | √ | √ | 23 |

**Abbreviations:** Item 1, Title; Item 2, Abstract; Item 3, Background and objectives; Item 4, Target population and subgroups; Item 5, Setting and location; Item 6, Study perspective; Item 7, Comparators; Item 8, Time horizon; Item 9, Discount rate; Item 10, Choice of health outcomes; Item 11, Measurement of effectiveness; Item 12, Measurement and valuation of preference-based outcomes; Item 13, Estimating resources and costs; Item 14, Currency, price date, and conversion; Item 15, Choice of model; Item 16, Assumptions; Item 17, Analytic methods; Item 18, Study parameters; Item 19, Incremental costs and outcomes; Item 20, Characterizing uncertainty; Item 21, Characterizing heterogeneity; Item 22, Study findings, limitations, generalization; Item 23, Source of funding; Item 24, Conflicts of interest. NA: not applicable.

Table 3. Quality assessment of studies with QHES instrument.

|  | Item 1 | Item 2 | Item 3 | Item 4 | Item 5 | Item 6 | Item 7 | Item 8 | Item 9 | Item 10 | Item 11 | Item 12 | Item 13 | Item 14 | Item 15 | Item 16 | Total scores |
| --- | --- | --- | --- | --- | --- | --- | --- | --- | --- | --- | --- | --- | --- | --- | --- | --- | --- |
| Wilson, 2005 | √ | × | √ | √ | √ | √ | √ | √ | × | √ | √ | √ | √ | √ | √ | √ | 88 |
| Huse, 2007 | √ | × | √ | √ | √ | √ | √ | √ | √ | √ | √ | √ | × | √ | √ | √ | 89 |
| Mabasa, 2008 | √ | √ | × | √ | √ | √ | √ | × | √ | √ | √ | √ | √ | √ | √ | × | 82 |
| Chabot, 2008 | √ | × | √ | √ | √ | √ | √ | √ | √ | √ | √ | √ | × | √ | √ | √ | 89 |
| Paz-Ares, 2008 | √ | × | √ | √ | √ | √ | √ | √ | √ | √ | √ | √ | √ | √ | √ | × | 93 |
| Contreras-Hernande, 2008 | √ | √ | √ | √ | √ | √ | √ | √ | √ | √ | √ | √ | √ | √ | √ | × | 97 |
| Hislop, 2011 | √ | × | √ | √ | √ | √ | √ | √ | √ | √ | √ | √ | √ | √ | √ | √ | 96 |
| Sanon, 2013 | √ | × | √ | √ | √ | √ | √ | √ | √ | √ | √ | √ | √ | √ | √ | √ | 96 |
| Majer, 2013 | √ | √ | √ | √ | √ | √ | √ | √ | √ | √ | √ | √ | √ | √ | √ | √ | 100 |
| Nerich, 2016 | √ | × | √ | √ | √ | √ | √ | √ | √ | √ | √ | √ | √ | √ | √ | √ | 96 |
| Tamoschus, 2017 | √ | √ | √ | √ | √ | √ | √ | √ | √ | √ | √ | √ | √ | √ | √ | √ | 100 |
| Bussabawalai, 2019 | √ | × | √ | √ | √ | √ | √ | √ | √ | √ | √ | √ | √ | √ | √ | √ | 96 |
| Zuidema, 2019 | √ | × | √ | √ | √ | √ | √ | √ | √ | √ | √ | √ | √ | √ | √ | × | 93 |
| Farid, 2020 | √ | × | √ | √ | √ | √ | √ | √ | √ | √ | √ | √ | √ | √ | √ | √ | 96 |
| Banerjee, 2020 | √ | × | √ | √ | √ | √ | √ | √ | √ | √ | √ | √ | √ | √ | √ | √ | 96 |

**Abbreviations:** Item 1, Study objective; Item 2, Perspective and reasons; Item 3, Variable source; Item 4, Prespecify subgroups; Item 5, Uncertainty handled; Item 6, Incremental analysis; Item 7, Methodology for data abstraction; Item 8, Time horizon and discount rate; Item 9, Measurement of costs and methodology for estimation; Item 10, Primary outcome measure; Item 11, Health outcomes measure; Item 12, Economic model, study methods and analysis; Item 13, Choice of economic model, main assumptions, and limitations; Item 14, Potential biases; Item 15, Conclusions based on the study results; Item 16, Statement disclosing the source of funding.
